# Abnormal Shift in B Memory Cell Profile Is Associated With the Expansion of Circulating T Follicular Helper Cells *via* ICOS Signaling During Acute HIV-1 Infection

**DOI:** 10.3389/fimmu.2022.837921

**Published:** 2022-02-10

**Authors:** Xiaofan Lu, Xin Zhang, Allen Ka Loon Cheung, Christiane Moog, Huan Xia, Zhen Li, Rui Wang, Yunxia Ji, Wei Xia, Zhiying Liu, Lin Yuan, Xiuwen Wang, Hao Wu, Tong Zhang, Bin Su

**Affiliations:** ^1^ Beijing Key Laboratory for HIV/AIDS Research, Sino-French Joint Laboratory for Research on Humoral Immune Response to HIV Infection, Clinical and Research Center for Infectious Diseases, Beijing Youan Hospital, Capital Medical University, Beijing, China; ^2^ Department of Biology, Faculty of Science, Hong Kong Baptist University, Hong Kong, Hong Kong SAR, China; ^3^ Laboratoire d’ImmunoRhumatologie Moléculaire, plateforme GENOMAX, INSERM UMR_S 1109, Institut Thématique Interdisciplinaire (ITI) de Médecine de Précision de Strasbourg, Transplantex NG, Faculté de Médecine, Fédération Hospitalo-Universitaire (FHU) OMICARE, Fédération de Médecine Translationnelle de Strasbourg (FMTS), Université de Strasbourg, Strasbourg, France

**Keywords:** B cells, T follicular helper cells, ICOS, IL-21, acute HIV-1 infection

## Abstract

Interactions between T follicular helper (Tfh) cells and germinal center B cells are essential for the differentiation of B cells and specific antibody responses against HIV-1 infection. However, the extent to which HIV-1 infection affects the dynamic interplay between these two cell populations in the bloodstream remains unclear. In this study, the dynamics of circulating Tfh (cTfh) and B cells and their relationship in individuals with acute and chronic HIV-1 infection were investigated. Twenty-five study subjects were enrolled from the Beijing PRIMO clinical cohort, a prospective cohort of HIV-1-negative men who have sex with men (MSM) for the identification of cases of acute HIV-1 infection (AHI) at Beijing Youan Hospital, Capital Medical University. Individuals with AHI were selected at random. Matched samples were also collected and analyzed from the same patients with chronic HIV-1 infection. None of the study subjects received antiretroviral therapy during acute or chronic infection. Multicolor flow cytometry was used for the immunophenotypic and functional characterization of cTfh cell and B cell subsets. AHI resulted in increased proportions in bulk cTfh, ICOS^+^cTfh or IL-21^+^ICOS^+^cTfh cells. In both acute and chronic infections, activated memory (AM), tissue-like memory (TLM), and plasmablast (PB) B cell levels were increased whilst resting memory (RM) and naïve mature (NM) B cell levels were decreased. Classical memory (CM) B cells were unaffected during infection. Association analyses showed that the levels of ICOS^+^cTfh and IL-21^+^ICOS^+^cTfh cells were negatively correlated with those of AM, CM, RM cells, and positively correlated with those of NM cells in AHI but not chronic HIV-1 infection stage (CHI). Moreover, the frequency of IL-21^+^ICOS^+^cTfh cells was also positively correlated with plasma HIV-1 viral load, and had an opposite association trend with CD4^+^T cell count in AHI. Our data suggests that HIV-1 infection drives the expansion of cTfh cells, which in turn leads to perturbations of B cell differentiation through ICOS signaling during acute infection stage. These findings provide insight on the role of ICOS in the regulation of cTfh/B cell interaction during AHI and may potentially guide the design of effective strategies for restoring anti-HIV-1 immunity in the infected patients.

## Introduction

Ineffective humoral responses is one of the hallmarks of HIV-1 infection (1). B cells are a key element of the adaptive immune system, and memory B cells are a subpopulation of B cells formed in germinal center (GC) after infection and are critical to induce long-lived humoral immune memory (2, 3). Memory B cells are characteristically impaired in HIV-1 patients. Resting memory (RM) subset, which is quite important for maintaining humoral responses, is decreased, whereas aberrant B cell subsets found in healthy individuals, active memory (AM), tissue-like memory (TLM), and plasmablasts (PB) are increased upon HIV-1 infection (4). TLM represents B cell exhaustion, and both of AM and PB are short-lived, unable to contribute to long-lasting humoral responses (5). All of these abnormal changes result in the lack of effective broadly neutralizing antibodies (bNAbs) in HIV-1 infected individuals (6, 7).

The establishment of protective humoral immune responses requires dynamic interaction between T follicular helper (Tfh) cells and B cells within the GC (8–10). *Bona fide* Tfh cells, predominantly express CXCR5, ICOS, PD-1, as well as Bcl-6, are an activated CD4^+^ T-cell subset specialized for providing help to B cell development in GC (11). Tfh-B cell interactions in the B cell follicles are responsible for GC formation, immunoglobulin (Ig) class-switching, differentiation of memory B cells, and antibody-secreting plasma cells in response to the secretion of IL-4, IL-9, IL-10, IL-21 by GC Tfh cells (12–16). ICOS expressed on GC Tfh cell surface is critical for their maintenance and functions (17–19). Deficiency of ICOS in mice results in the defective GC formation as well as impaired antibody responses (17, 19). Expansion of the GC Tfh cell population and the impairment of Tfh-B cell interactions during chronic HIV-1 infection (CHI) led to a profound dysfunction of the humoral immune response, including hypergammaglobulinemia, polyclonal B cell activation, and the disruption of B cell differentiation, all of which can affect the ability of HIV-1-infected individuals to develop bNAbs (20–23). Mechanistic studies revealed that inadequate Tfh cell functions resulted in impaired B cell immunity during HIV-1 infection, due to the PD-1-PD-L1 interaction between Tfh and B cells in infected lymph nodes (23, 24).

Unlike the GC Tfh cells, circulating Tfh (cTfh) cells have a memory phenotype, with lack of Bcl-6 expression (25, 26). Several lines of evidence suggest that cTfh cells can also deliver help for B cell maturation and neutralizing antibody generation after virus infection, such as HIV/SIV, COVID-19, or vaccination (19, 20, 27–32). Chronic HIV-1 infection compromises both the number and function of cTfh cells and damages cTfh-B cell interactions, posing obstacles to mounting an effective humoral response against HIV-1 (27, 33–38). The preservation of cTfh cells is associated with a strong HIV-1-specific B cell response or the broader range of bNAbs in HIV-1 elite controllers or individuals with CHI (6, 39–41).

The dynamic interplay between GC Tfh and B cells had been crucial for an effective humoral immune response during HIV-1 infection (21). However, the changes in cTfh cell levels during acute HIV-1 infection (AHI) is unclear, their effect on B cell differentiation and the underlying mechanisms have yet to be characterized. Alterations in B cell subpopulations in AHI have been reported (4, 42). We therefore conducted a cohort study to trace the changes in cTfh and B cell levels during the acute and chronic stages of HIV-1 infection, and the association between these two cell populations. Here, we show evidence that ICOS^+^cTfh cells are related for the abnormal B cell development in HIV-1 acute infection stage. The study findings add to our understanding of the fate and dynamics of cTfh cells and circulating B cells during HIV-1 infection may provide potential new strategies for protective HIV-1 vaccination or treatments.

## Methods and Materials

### Study Subjects

The study participants were enrolled from the Beijing PRIMO cohort of men who have sex with men (MSM) and are at high risk of HIV-1 infection (43). They were screened every two months for acute HIV-1 infection at Beijing Youan Hospital. Acute infection was defined as a positive result for the detection of HIV-1 RNA but a negative or indeterminate result for the detection of anti-HIV-1 antibodies. Once acute infection was detected, patients were followed at 1, 2, 4, 8, and 12 weeks, and every three months thereafter, with determinations of CD4^+^ T-cell counts, viral load determinations and syphilis status. Chronic infection was considered to begin six months after the diagnosis of acute infection. Blood samples were collected at multiple time points as mentioned above. Peripheral blood mononuclear cells (PBMCs) were isolated from patients and frozen with liquid nitrogen until used. 25 individuals with acute HIV-1 infection were enrolled in this study. We analyzed both acute-phase and consequent chronic-phase samples (at least 1 year after acute infection) from these individuals. All individuals with AHI were classified as having Fiebig stage III–VI disease after the diagnosis of HIV-1 infection. None of the study subjects received ART during the acute or chronic stage of infection. Individuals with opportunistic infections, tuberculosis, autoimmune diseases or HBV/HCV co-infection were excluded. Detailed information about the patient samples studied is provided in **Table 1**. We enrolled 21 age-matched HIV-1-negative individuals from the MSM population with high-risk behavior as healthy controls.

**Table 1:** Characteristics of HIV-1-infected individuals.

HIV-1-infected individuals	Acute HIV-1 infection stage (AHI)			Chronic HIV-1 infection stage (CHI)		
	Estimated infection days	CD4 counts (cells/μl)	Viral loads (copies/ml)	Estimated infection days	CD4 counts (cells/μl)	Viral loads^*^ (copies/ml)
1	37	571	438	598	243	/
2	75	429	4880	431	244	/
3	40	636	22200	427	254	/
4	40	508	N.D.^#^	528	97	/
5	65	498	384996	370	230	/
6	60	658	119055	433	209	/
7	80	533	105797	731	279	/
8	52	504	15135	534	225	/
9	41	683	1734	243	283	/
10	34	482	20404	251	152	/
11	94	496	25303	258	211	/
12	38	478	1353187	174	233	/
13	65	673	1610	392	309	/
14	68	599	114992	743	564	/
15	56	419	158272	743	469	/
16	50	665	14517	634	419	/
17	41	629	2380	706	490	/
18	79	688	15030	605	579	/
19	75	498	258000	175	251	/
20	34	525	201554	323	210	/
21	92	553	8387	244	241	/
22	85	755	3280	344	511	/
23	78	496	6669	862	537	/
24	54	683	40858	258	415	/
25	41	600	464396	666	420	/

^*^ /, viral loads are not determined in CHI;

^#^ N.D., viral loads are not determined.

### Immune Phenotyping

Cryopreserved PBMCs were thawed, and stained with Live/Dead fixable viability stain 510 or 620 for 15 min to exclude dead cells. Cells were then washed, and surface stained with anti-human mAbs at room temperature for 20 min in the dark. Antibodies used for cell surface staining included: CD3-BV650 (Clone UCHT1), CD4-FITC (Clone OKT4), CD4-PE-Cy7 (Clone OKT4), CD10-FITC (Clone HI10a), CD27-PerCP-Cy5.5 (Clone O323), CD19-APC (Clone HIB19), CXCR3-APC-Cy7 (Clone G025H7), CD20-APC-Cy7 (Clone 2H7), CD21-BV421 (Clone B-ly4), PD-1-BV421 (Clone EH12.1), CXCR5-AF647 (Clone J252D4), CXCR5-FITC (Clone J252D4), CD278 (ICOS)-PE (Clone C398.4A), and CD154 (CD40L)-PE-Cy7 (Clone 24-31). Samples were washed before data acquisition on a BD LSR Fortessa flow cytometer with Diva software (BD Biosciences). For Ki-67 staining, after surface staining with appropriate antibodies, cells were permeabilized and fixed with intracellular staining reagents according to the manufacturer’s instructions (eBioscience, San Diego, CA, USA) before intracellular staining with anti-Ki-67-PE-Cy7 (Clone Ki-67) mAb. The corresponding isotype controls or fluorescence minus one (FMO) sample were prepared to facilitate gating and signals. All antibodies and isotype controls were purchased from BD Biosciences or Biolegend Company (San Diego, CA, USA).

**Figure 1:**
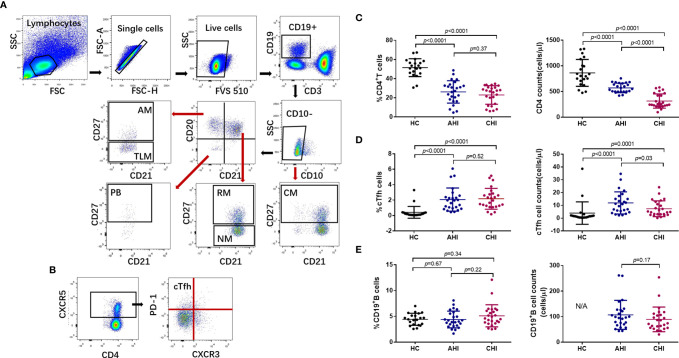
Expansion of bulk cTfh cells after HIV-1 acute infection and persists into chronic stage. **(A)** The gating strategy for flow cytometric analysis of bulk B cell and B cell subsets in PBMCs. Among all events, forward angle and side scatter light gating were gated on lymphocytes and were used to exclude cell debris from the analysis. Forward height and forward area were used to exclude doublet cells, and cells were labeled with Live/Dead fixable viability stain 510, and dead cells were excluded. Bulk B cells were defined as CD19^+^CD10^-^, and were classified as classical memory (CM, CD27^+^), activated memory (AM, CD20^+^CD21^-^CD27^+^), tissue-like memory (TLM, CD20^+^CD21^-^CD27^-^), resting memory (RM, CD20^+^CD21^+^CD27^+^), naïve (NM, CD20^+^CD21^+^CD27^-^), and plasmablasts (PB, CD20^-^CD21^-^CD27^+^). FSC, forward scatter; SSC, side scatter. **(B)** Representative analysis of cTfh cells (CD3^+^CD4^+^CXCR3^-^CXCR5^+^PD-1^+^). **(C–E)** Comparison of the frequencies (*left*) and number (*right*) of bulk CD4^+^T cells, cTfh cells, and B cells among healthy control (HC, n=21), acute HIV-1 infection stage (AHI, n=25), and chronic HIV-1 infection stage (CHI, n=25) groups. All data shown are means ± SD. N/A, data not available.

Cytometer setup and tracking (CST) calibration particles were used to ensure that fluorescence intensity measurements were consistent in all experiments. UltraComp eBeads (eBioscience, San Diego, CA, USA) were used for compensation. Gating on forward scatter (FSC) and side scatter (SSC) light was used to exclude cell debris from the analysis; forward height and forward area were used to exclude doublet cells. At least 2×10^5^ live cells were acquired per sample. Data were analyzed with Flowjo Software version 10 (Tree Star Inc., Ashland, OR, USA). The gating strategies for the analysis of flow cytometry data are described in **Figure 1**. Bulk CD19^+^B cells were defined as CD19^+^CD10^-^, and were further classified into the following six subsets on the basis of CD20, CD21, and CD27 expression: classical memory (CM, CD27^+^), activated memory (AM, CD20^+^CD21^-^CD27^+^), tissue-like memory (TLM, CD20^+^CD21^-^CD27^-^), resting memory (RM, CD20^+^CD21^+^CD27^+^), naïve mature (NM, CD20^+^CD21^+^CD27^-^), and plasmablasts (PB, CD20^-^CD21^-^CD27^+^) (**Figure 1A**) (44, 45). cTfh cells were defined as CD3^+^CD4^+^CXCR3^-^CXCR5^+^PD-1^+^ (**Figure 1B**) (29).

### Intracellular IL-21 Staining

PBMCs were stimulated by leukocyte activation cocktail with BD GolgiPlug (BD Biosciences) in an incubator at 37°C with 5% CO_2_ for 6 h. The leukocyte activation cocktail contained phorbol 12-myristate-13-acetate (PMA), ionomycin, and brefeldin A. After stimulation, cells were washed and stained with cTfh cell surface phenotype panel for 20 min at room temperature in the dark. After permeabilization and fixation, cells were intracellularly stained with anti-human-IL-21-PE mAb (Clone 3A3-N2) (Biolegend). Data were acquired and analyzed as described above.

### ELISA

The IL-21 ELISA MAX kit (Biolegend) was used for quantification of IL-21 concentration in plasma samples, according to the manufacturer’s instructions.

### CD4^+^T Cell Count and Viral Load Measurement

Routine blood CD4^+^ T cell counts (cells/μl) were obtained by four-color flow cytometry with human CD45^+^, CD3^+^, CD4^+^ and CD8^+^ cell markers (BD Biosciences), on peripheral whole-blood samples from each patient, in FACS lysing solution (BD Biosciences), according to the manufacturer’s instructions. Plasma HIV-1 viral load (copies/ml of plasma) was quantified by real-time PCR assay (Abbott Molecular Inc., Des Plaines, IL, USA) with a sensitivity of 40 copies/ml of plasma for viral RNA detection.

### Statistical Analysis

Data are expressed as means ± standard deviation (SD). Statistical analysis was performed with GraphPad Prism software version 5 (GraphPad Software, San Diego, California, USA). Differences were analyzed with nonparametric two-tailed Mann-Whitney tests. Spearman’s rank correlation analyses were performed to assess relationships between two variables. Differences were considered statistically significant if *p*<0.05 in two-tailed tests.

### Ethics Statement

This study and all the relevant experiments were approved by the Beijing Youan Hospital Research Ethics Committee (No. 2018-035), and written informed consent was obtained from each participant in accordance with the Declaration of Helsinki. The clinical samples collected were stored and used for research. The methods used conformed to approved guidelines and regulations.

## Results

### Acute HIV-1 Infection Drives cTfh Cell Expansion and B Cell Subset Perturbation

We investigated the dynamics of cTfh cells and B cells during HIV-1 infection by flow cytometry according to the gating strategy shown in **Figure 1**. CD4^+^ T cells were first quantified in samples from three groups: uninfected individuals (healthy controls, HC, n=21), individuals with acute-phase HIV-1 infection (AHI, n=25) and matched individuals with chronic-phase infection (CHI, n=25). As expected, HC group had the highest CD4^+^T cell proportion (AHI *vs* HC: 26.22 ± 12.15 *vs* 51.46 ± 9.15, *p*<0.0001; CHI *vs* HC: 21.48 ± 9.59 *vs* 51.46 ± 9.15, *p*<0.0001; AHI *vs* CHI: 26.22 ± 12.15 *vs* 21.48 ± 9.59, *p*=0.37, **Figure 1C**, *left*) and cell numbers (AHI *vs* HC: 567 ± 89 *vs* 862 ± 254, *p*<0.0001; CHI *vs* HC: 276 ± 110 *vs* 862 ± 254, *p*<0.0001; AHI *vs* CHI: 567 ± 89 *vs* 276 ± 110, *p*<0.0001, **Figure 1C**, *right*) among three groups, both AHI and CHI groups were dramatically decreased. There is currently no consensus concerning the most appropriate phenotypic markers for cTfh cells, and the combinations of cell surface markers used to identify bulk cTfh cells, including CXCR5, CXCR3, CCR6, PD-1, IL-21, and ICOS, differ between studies. Locci et al. showed that PD-1^+^CXCR3^-^CXCR5^+^ cTfh cells are the counterpart of GC Tfh cells, as they have a similar gene expression, cytokine profile and functionalities to GC Tfh cells, and are correlated with the breadth of bNAbs in HIV-1-infected individuals (29). We therefore considered PD-1^+^CXCR3^-^CXCR5^+^CD4^+^ T cells to be the bulk cTfh cells (**Figure 1B**). Phenotypic analysis showed that the frequency of bulk cTfh cells rose significantly in AHI and CHI groups, compared to HC (AHI *vs* HC: 1.89 ± 1.45 *vs* 0.41 ± 0.74, *p*<0.0001; CHI *vs* HC: 1.97 ± 1.27 *vs* 0.41 ± 0.74, *p*<0.0001; AHI *vs* CHI: 1.89 ± 1.45 *vs* 1.97 ± 1.27, *p*=0.52, **Figure 1D**, *left*). The bulk cTfh cell count was highest in AHI, and declined during CHI due to the loss of total CD4 count, though remained higher than HC (AHI *vs* HC: 11 ± 8 *vs* 4 ± 8, *p*<0.0001; CHI *vs* HC: 5 ± 4 *vs* 4 ± 8, *p*=0.0001; AHI *vs* CHI: 11 ± 8 *vs* 5 ± 4, *p*=0.03, **Figure 1D**, *right*). This result indicates that the expansion of cTfh cells occurred since primary infection and maintained into CHI.

**Figure 2:**
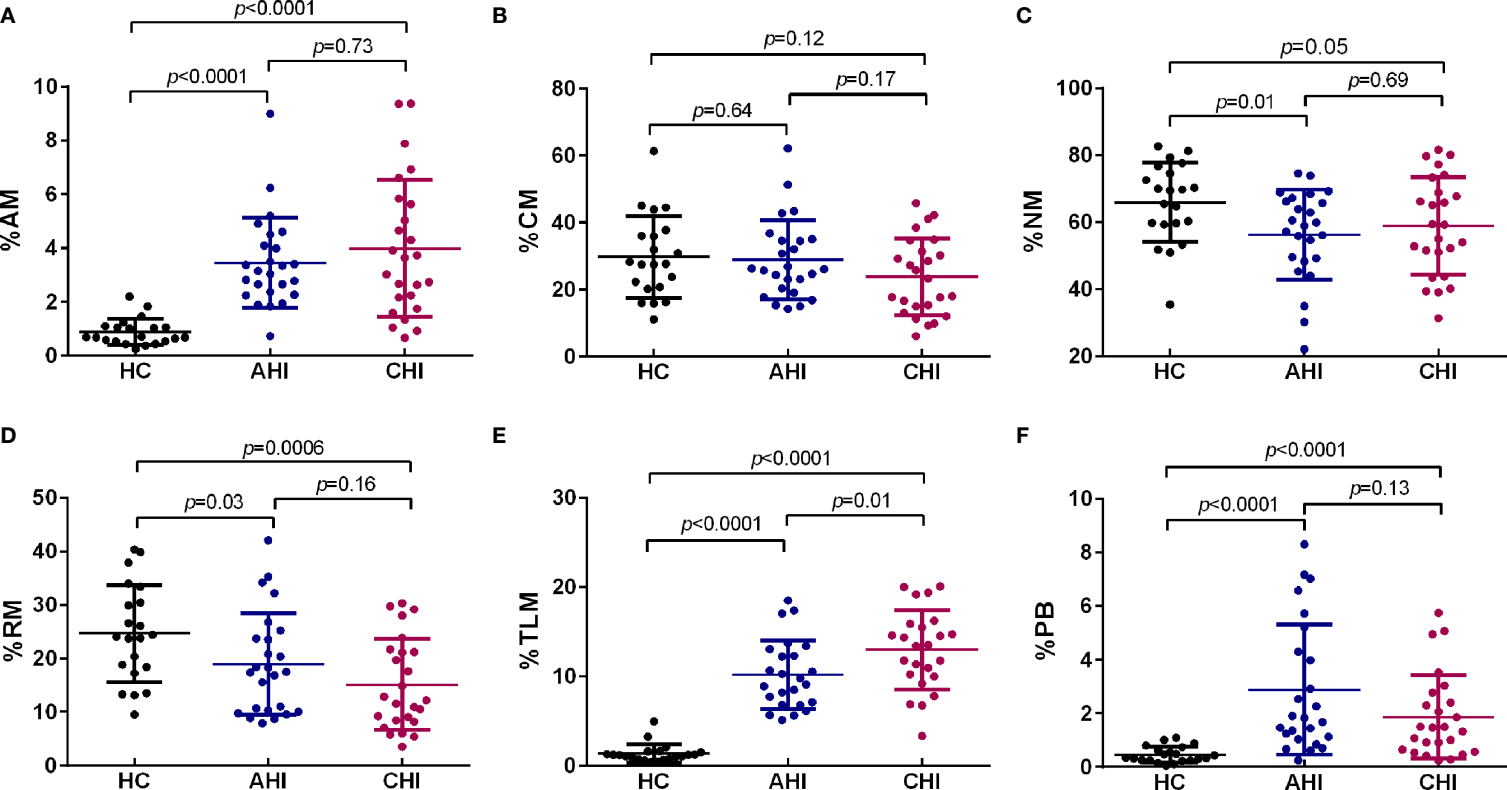
HIV-1 acute infection drives B cell subsets perturbation. Comparison of AM **(A)**, CM **(B)**, NM **(C)**, RM **(D)**, TLM **(E)**, and PB **(F)** among HC (n=21), AHI (n=25), and CHI (n=25) groups. AM, activated memory B cells; CM, classical memory B cells; NM, naive B cells; RM, resting memory B cells; TLM, tissue-like memory B cells; PB, plasmablasts. All data shown are means ± SD.

Analysis of B cells revealed that the percentages of bulk CD19^+^B cells in the individuals of the AHI and CHI groups were similar compared to uninfected individuals (AHI *vs* HC: 4.32 ± 1.60 *vs* 4.42 ± 1.14, *p*=0.67; CHI *vs* HC: 5.20 ± 2.20 *vs* 4.42 ± 1.14, *p*=0.34; AHI *vs* CHI: 4.32 ± 1.60 *vs* 5.20 ± 2.20, *p*=0.22, **Figure 1E**, *left*). CD19^+^B cell counts was lower in CHI than in AHI, although the statistics did not reach significance (AHI *vs* CHI: 104 ± 58 *vs* 88 ± 51, *p*=0.17, **Figure 1E**, *right*). Further, statistical analyses for each B cell subset in the three groups found that the AHI and CHI groups had higher frequencies of AM, TLM, PBs and lower frequencies of RM and NM than the HC group (AM: AHI *vs* HC: 3.35 ± 1.73 *vs* 0.87 ± 0.47, *p*<0.0001; CHI *vs* HC: 3.83 ± 2.42 *vs* 0.87 ± 0.47, *p*<0.0001; TLM: AHI *vs* HC: 10.25 ± 3.87 *vs* 1.39 ± 1.00, *p*<0.0001; CHI *vs* HC: 12.97 ± 4.45 *vs* 1.39 ± 1.00, *p*<0.0001; PB: AHI *vs* HC: 2.41 ± 2.07 *vs* 0.46 ± 0.28, *p*<0.0001; CHI *vs* HC: 1.73 ± 1.60 *vs* 0.46 ± 0.28, *p*<0.0001; RM: AHI *vs* HC: 19.67 ± 9.66 *vs* 24.69 ± 8.85, *p*=0.03; CHI *vs* HC: 14.63 ± 8.01 *vs* 24.69 ± 8.85, *p*=0.0006; NM: AHI *vs* HC: 56.64 ± 13.81 *vs* 65.93 ± 11.53, *p*=0.01; CHI *vs* HC: 59.40 ± 14.01 *vs* 65.93 ± 11.53, *p*=0.05). By contrast, CM frequency was similar in all three groups (**Figure 2**). Besides, a higher proportion of TLM in CHI than that in AHI was found (AHI *vs* CHI: 10.25 ± 3.87 *vs* 12.97 ± 4.45, *p*=0.01), however, no significant difference was observed between the AHI and CHI groups for the other B cell subsets (**Figure 2**). Thus, although the observation of preserved bulk CD19^+^ B cells was shown in both AHI and CHI, altered B cell subset proportions also occurred during AHI and persisted into CHI.

### Higher ICOS Expression on cTfh Cells Is Associated With Abnormal Memory B Cell Differentiation During Acute HIV-1 Infection

**Figure 3:**
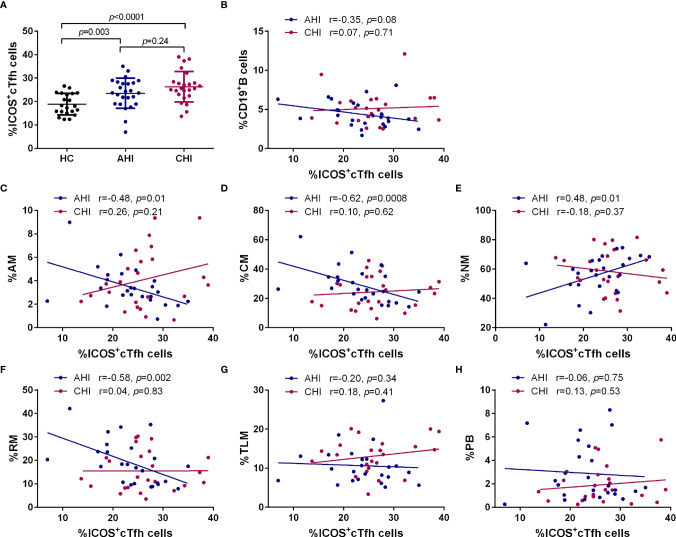
Correlation between the frequencies of B cell subsets and ICOS^+^cTfh cells in the AHI and CHI groups. **(A)**. The frequency of ICOS^+^cTfh cells between HC (n=21), AHI (n=25), and CHI (n=25). All data shown are means ± SD. **(B–H)**. Correlation analysis of the ICOS^+^cTfh cell frequency with bulk B cells, AM, CM, NM, RM, TLM, and PB in the AHI and CHI groups.

Since ICOS and CD40L are two essential costimulatory molecules for Tfh cell maintenance and Tfh/B cell interaction in GC, we next investigated these two molecules in cTfh cells as well as the effect on B cell differentiation. Compared to HC, both ICOS (AHI *vs* HC: 23.76 ± 6.60 *vs* 18.83 ± 4.43, *p*=0.003; CHI *vs* HC: 26.13 ± 6.94 *vs* 18.83 ± 4.43, *p*<0.0001, **Figure 3A**) and CD40L (AHI *vs* HC: 22.26 ± 3.01 *vs* 15.55 ± 5.95, *p*<0.0001; CHI *vs* HC: 23.08 ± 3.63 *vs* 15.55 ± 5.95, *p*<0.0001, **Supplementary Figure 1A**) expression level on cTfh cells were elevated during AHI stage and persisted into CHI stage. However, there was no significant difference between AHI and CHI, although CHI had a slightly higher level of ICOS and CD40L on cTfh cells.

Next, we performed linear regression analysis between ICOS^+^cTfh or CD40L^+^cTfh cells and bulk CD19^+^B cells or different B cell subsets during AHI and CHI groups. The frequency of ICOS^+^cTfh cells was not correlated with bulk CD19^+^ B cells in AHI (r=-0.35, *p*=0.08) and CHI (r=0.07, *p*=0.71) (**Figure 3B**). Interestingly, strong correlations were observed for ICOS^+^cTfh and B cell subsets (except TLM and PB) in AHI. Briefly, as AM subset was increased during AHI, they were found to be negatively correlated with ICOS^+^cTfh cells (r=-0.48, *p*=0.01, **Figure 3C**). NM and RM subsets that were decreased upon HIV-1 exposure, were positively (r=0.48, *p*=0.01) and negatively (r=-0.58, *p*=0.002) correlated, respectively, with ICOS^+^cTfh cell frequency during AHI (**Figures 3E, F**). CM that was presented at similar frequencies in all three groups, was found negatively associated with AHI ICOS^+^cTfh cells (r=-0.62, *p*=0.0008, **Figure 3D**). In contrast, similar associations between ICOS^+^cTfh cells and B cell subsets were not observed during CHI stage. No relationship was observed between TLM or PBs and ICOS^+^cTfh cells in both AHI and CHI, even though these two subsets were significantly expanded after HIV-1 infection (**Figures 2E, F**). ICOS^-^cTfh cells had no relationship with B cell subsets (data not shown). Also, no correlation was found between CD40L^+^cTfh cells and bulk CD19^+^B cells or any B cell subset during AHI or CHI stage (**Supplementary Figures 1B-H**).

ICOS is expressed on activated T cells (46, 47), therefore, we analyzed the expression level of cell cycling marker Ki-67 on cTfh cells. Expectedly, the Ki-67 expression on cTfh cells was significantly increased upon HIV-1 infection (AHI *vs* HC: 11.85 ± 5.18 *vs* 7.79 ± 2.15, *p*=0.01; CHI *vs* HC: 14.23 ± 6.82 *vs* 7.79 ± 2.15, *p*<0.0001, **Supplementary Figure 2A**). The percentage of Ki-67^+^cTfh cells had strong correlations with ICOS^+^cTfh cells (AHI: r=0.68, *p*=0.0002; CHI: r=0.52, *p*=0.008; **Supplementary Figure 2B**), but not with CD40L^+^cTfh cells during both AHI and CHI stages (**Supplementary Figure 2C**). Therefore, these results indicated that ICOS^+^cTfh cells are likely functional and activated by acute HIV-1 infection, and could be involved in the impairment of interacting with B cells, and thereby influenced on memory B cell differentiation.

### IL-21 Secreting ICOS^+^cTfh Cells Contribute to the Memory B Cell Subsets Imbalance During Acute HIV-1 Infection

IL-21 is known to be a hallmark cytokine secreted by Tfh cells and plays an important role in B cell development and antibody generation (28, 48, 49). Therefore, we evaluated the plasma IL-21 level and IL-21-producing bulk cTfh cells. Unexpectedly, no differences were found among AHI, CHI, and HC in plasma IL-21 level (**Supplementary Figure 3A**). By intracellular flow cytometric analysis, IL-21^+^bulk cTfh cells was lower in both AHI and CHI than HC (AHI *vs* HC:7.65 ± 4.64 *vs* 10.41 ± 5.42, *p*=0.04; CHI *vs* HC: 6.97 ± 4.68 *vs* 10.41 ± 5.42, *p*=0.009, **Supplementary Figure 3B**). Moreover, the frequency of IL21^+^cTfh positively correlated with plasma IL-21 levels in AHI (r=0.38, *p*=0.05) but not in CHI (**Supplementary Figure 3C**). However, no association was observed between the frequency of IL21^+^cTfh and bulk CD19^+^B or any B cell subsets (**Supplementary Figures 3E-J**).

**Figure 4:**
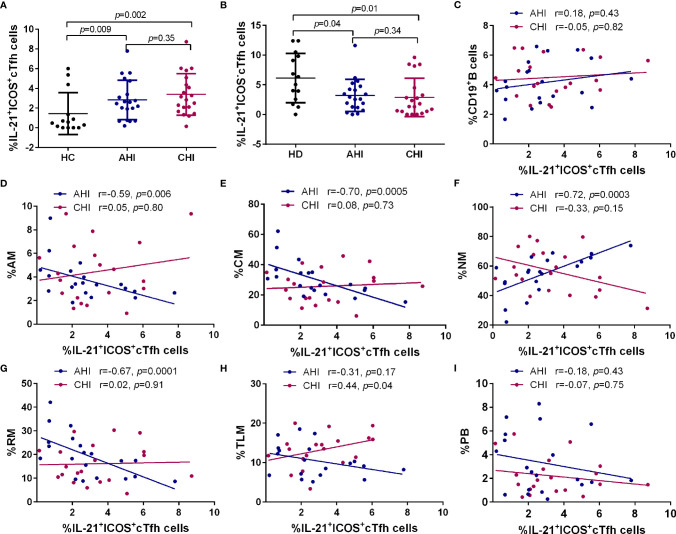
Correlation between the frequencies of B cell subsets and IL-21^+^ICOS^+^cTfh cells in the AHI and CHI groups. PBMCs from HC (n=13), AHI (n=20) and CHI (n=20) were stimulated with leukocyte activation cocktail for 6 h *in vitro* and the frequency of IL-21^+^ICOS^+^cTfh cells **(A)** or IL-21^+^ICOS^-^cTfh cells **(B)** was measured by flow cytometry. All data shown are means ± SD. **(C–I)**. Correlation analysis of the IL-21^+^ICOS^+^cTfh cell frequency with bulk B cells, AM, CM, NM, RM, TLM, and PB in the AHI and CHI groups.

Since strong correlations between ICOS^+^cTfh cells and memory B cell subsets were found in AHI, we next determined the level of IL-21 secretion by ICOS^+^cTfh cells and their association with B cell subsets in different groups. As shown in **Figure 4A**, compared to HC, the percentage of IL-21 within ICOS^+^cTfh cells was elevated in AHI and CHI groups (AHI *vs* HC:2.87 ± 1.96 *vs* 1.44 ± 2.04, *p*=0.009; CHI *vs* HC: 3.40 ± 2.16 *vs* 1.44 ± 2.04, *p*=0.002). By contrast, the level of IL-21 by ICOS^-^cTfh cells was declined in AHI and CHI groups (AHI *vs* HC:3.41 ± 2.91 *vs* 6.13 ± 4.00, *p*=0.04; CHI *vs* HC: 2.52 ± 2.95 *vs* 6.13 ± 4.00, *p*=0.01, **Figure 4B**). Linear regression analysis revealed that the association between IL-21^+^ICOS^+^cTfh cells and bulk CD19^+^B cells or different B cell subset in AHI was similar to those with ICOS^+^cTfh cells as above. Briefly, the frequency of IL-21^+^ICOS^+^cTfh cells was positively correlated with NM (r=0.72, *p*=0.0003, **Figure 4F**), and negatively correlated with AM (r=-0.59, *p*=0.006, **Figure 4D**), CM (r=-0.70, *p*=0.0005, **Figure 4E**), and RM (r=-0.67, *p*=0.0001, **Figure 4G**) in AHI. Similar associations between IL-21^+^ICOS^+^cTfh cells and B cell subsets were not found in CHI, except for weak positive correlation between IL-21^+^ICOS^+^cTfh cells and TLM in CHI (r=0.44, *p*=0.04) but not in AHI (**Figure 4H**). Neither bulk CD19^+^B cells nor PBs were associated with IL-21^+^ICOS^+^cTfh cells in both AHI and CHI (**Figures 4C, I**). Besides, no correlation was observed between IL-21^+^ICOS^-^cTfh cells and bulk CD19^+^B cells or any B cell subset in our study (data not shown). Thus, the increased level of IL-21^+^ICOS^+^cTfh cells may have effect on the mediation of B cell help by cTfh cells.

### IL-21^+^ICOS^+^cTfh Cells Are Involved in Disease Progression

**Figure 5:**
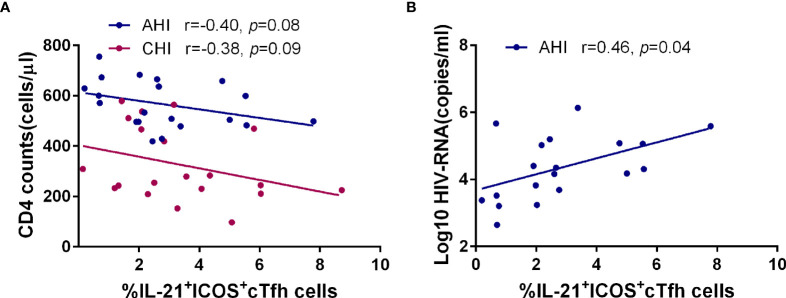
The frequency of IL-21^+^ICOS^+^cTfh cells in AHI is associated with the loss of CD4^+^T cells and high plasma viral load. Correlation analysis of the frequency of IL-21^+^ICOS^+^cTfh cells with CD4^+^T cell number **(A)** and plasma HIV-1 viral load **(B)** in the AHI (n=20) and CHI groups (n=20).

We also investigated the relationship between cTfh cells and CD4^+^T cell count or plasma HIV-1 viral load after HIV-1 infection. As shown in **Figure 5**, the frequency of IL-21^+^ICOS^+^cTfh cells had a negative correlation trend with CD4^+^T cell count in both AHI (r=-0.40, *p*=0.08) and CHI (r=-0.38, *p*=0.09) (**Figure 5A**), and positive correlation with viral load in AHI (r=0.46, *p*=0.04, **Figure 5B**). We did not perform the correlation analysis between IL-21^+^ICOS^+^cTfh cells and viral load in CHI due to the lack of viral load data in CHI. Similar relationship was not observed in bulk cTfh cells or ICOS^+^cTfh cells. These results suggested that IL-21^+^ICOS^+^cTfh cell population may contribute to HIV-1 disease progression.

## Discussion

Humoral immunity is considered an important component of effective vaccination against HIV-1 by giving rise to neutralizing antibodies that can eliminate viruses during both acute and chronic HIV-1 infections (50), as characterized by the activation of B lymphocytes and their progeny (3, 51). Cognate interactions between B cells and Tfh cells in the GC regions results in the differentiation of long-lived memory B cells and plasma cells, that are responsible for effective humoral responses and bNAbs (11).

The accumulation of Tfh cells during HIV-1 infection and impairment of Tfh-B cell interactions in GCs and the effect on humoral responses are well described in recent decades, but the fate of cTfh cells after HIV-1 infection remains controversial. Some studies observed that the frequency of cTfh cells were high in HIV-1 infected individuals (52, 53), which was consistent with our findings (**Figure 1D**). cTfh cells are highly permissive to HIV-1 (54, 55), where viral replication or antigen stimulation may drive the proliferation and accumulation of cTfh cells. Cytokine storm in AHI, such as elevated IL-6 and IFN-γ during HIV/SIV infection, could be potential mechanisms for the expansion of cTfh cells (56, 57). Other groups reported a similar or diminished cTfh cells in HIV-1 infected patients compared to healthy individuals (33, 35, 36, 38, 58). Explanations for the conflicting findings for the change of cTfh cells frequency during HIV-1 infection could be the different choices of markers used for the analysis of cTfh cells or differences between infection phases. Most previous studies have focused on chronic HIV-1 infection, whereas we found that the level of cTfh cells was already increased in acute infection stage.

ICOS and CD40L are two key secondary costimulatory molecules involved in Tfh/B collaboration. Engagement of ICOS in Tfh cells with its ligand (ICOSL) expressed on B cell surface is responsible for the regulation of Tfh differentiation and GC formation through PI3K signaling, whereas the CD40/CD40L interaction between Tfh/B promotes the Ig class-switching *via* NF-кB pathway (11, 59, 60). Consistent with previous studies, our data showed a loss of RM as well as the expansion of AM, TLM, and PBs occurred in HIV-1 acute infected stage and persisted into chronic stage (**Figure 2**) (61). Of note, the associated relationship with the B cell subset perturbation was only observed in the frequency of ICOS^+^cTfh cells or that of IL-21^+^ ICOS^+^cTfh cells in AHI but not in CD40L^+^ cTfh cells in both AHI and CHI in our study (**Figures 3**, **4** and **Supplementary Figure 1**). It seems likely that ICOS rather than CD40L signaling is more critical for the disruption of B cell development by cTfh cells during HIV-1 infection, especially in acute infection stage. ICOS signaling on GC Tfh is important for Tfh maintenance and GC formation as well as B cell differentiation (17–19). Rasheed et al. reported that only ICOS^hi^CXCR5^hi^CD4^+^T cells in tonsils can secrete high level of IL-21 as well as CXCL13, which are the main inducers for B cell maturation and Ig production, suggesting that GC Tfh with high level of ICOS expression is a terminally differentiated subset with efficient B helper activity (18). Several studies show that the induction of ICOS^+^cTfh or IL-21^+^ICOS^+^cTfh cells in blood is correlated with heightened antigen-specific antibody titer or humoral immune responses to influenza vaccination in healthy cohorts or in HIV-1-infected patients (49, 62–64). ICOS is expressed on activated cell surface (46, 47), therefore, above reports indicate that although cTfh cells have a memory phenotype in circulating, they still require to be activated to fulfill their helper function for B cells. Our study observed a positive relationship between IL-21^+^ICOS^+^cTfh cells and plasma HIV-1 viral load in acute infection stage (**Figure 5B**), also implicated that the cTfh cells are activated and exerted their IL-21 secretion capacity upon HIV-1 stimulation.

On the contrary, higher level of ICOS expression on cTfh cells also has side effect to B cell differentiation and antibody production. In systemic lupus erythematosus (SLE), expansion of ICOS^+^cTfh cells has been associated with the disturb of B cell development and higher autoantibody production (65, 66). Besides, the level of donor-specific anti-HLA antibodies (DSAs) is correlated with the frequency of CXCR5^+^PD-1^+^ICOS^+^cTfh cells after renal transplantation, thereby influencing the clinical outcome (67). IL-21 is a key B cell helper cytokine (13). Although the IL-21 secretion ability by bulk cTfh cells was reduced in both acute and chronic infection stage in our study, no relationship was observed between IL-21^+^cTfh cells with any memory B cells and PBs (**Supplementary Figure 3**). However, the correlation between the elevated level of ICOS^+^cTfh or IL-21^+^ICOS^+^cTfh cells with memory B cell subsets during acute HIV-1 infection stage in our results (**Figures 3**, **4**) indicated that signaling *via* ICOS after cTfh activation may drive the memory B cell perturbation as early as primary infection stage. The expansion of TLM and PBs may be the synergism of ICOS^+^cTfh or IL-21^+^ICOS^+^cTfh cells with other factors, such as immune activation and chronic inflammation after HIV-1 infection. Such effects on B cell subset differentiation, as shown by our data, may result in the dysfunction of humoral immune responses in HIV-1 patients, including non-specific B cell proliferation and hypersecretion of IgG1 antibody, all of which induce hypergammaglobulinemia (68, 69). In addition, Lu et al. demonstrated that high level of ICOS^+^cTfh cells may accelerate the CD4^+^T cell depletion in HIV-1 infected individuals (53). We also observed a similar result that the negative association trends between the frequency of IL-21^+^ICOS^+^cTfh cells and CD4^+^T cell count in both acute and chronic HIV-1 infection stage (**Figure 5A**). Our previous study demonstrated that higher level of anti-CD4 antibody is responsible for CD4^+^T cell depletion during HIV-1 acute infection (70). Therefore, we hypothesize that the overactivation of cTfh cells with high ICOS level and more IL-21 secretion by ICOS^+^cTfh cells induces the excessive anti-CD4 antibody generation, thereby promoting the progression of CD4^+^T cell loss even the induction of idiopathic CD4 lymphopenia (71).

This study faced several limitations. Firstly, the sample size is relatively small, which may have resulted in a deviation of the experimental data. Secondly, we did not take GC Tfh cells into account in this study. Larger samples and more detailed physiologically relevant studies are therefore required to address these issues in depth. Moreover, further studies are required to investigate the distribution of HIV-1-specific antibody responses between memory B cell subsets. Thirdly, the functional aspects of cTfh cells should be investigated *ex vivo* from acute and chronic patients. However, it is hard to sort cTfh cells from PBMCs due to the several markers used in our study to identify cTfh cells.

Overall, we found marked expansions of bulk cTfh, ICOS^+^cTfh and IL-21^+^ICOS^+^cTfh cell frequencies, as well as aberrant alterations to memory B cell subsets in peripheral blood occurred during the acute stage of HIV-1 infection, and these abnormalities persisted into chronic infection, consistent with the B cell dysfunction observed in HIV-1 disease. Moreover, the altered memory B cell subset distribution was correlated with changes in the frequencies of ICOS^+^cTfh and IL-21^+^ICOS^+^cTfh cells during AHI, suggesting that the memory B cell subset profile changes could be a result of cTfh cells overactivation mediated by pathway of ICOS signaling at the early stages of HIV-1 infection. Collectively, our findings provide valuable insights into the kinetics of cTfh cells and the underlying mechanisms that association between cTfh and B cells in AHI that deepen our understanding of HIV-1 pathogenesis. In addition, the abnormal cTfh cells and B cell subset distribution found in patients with AHI strengthened the importance of the initiation of antiretroviral therapy at the early stage of HIV-1 infection.

## Data Availability Statement

The data for this study are available by contacting the corresponding authors upon reasonable request.

## Ethics Statement

The studies involving human participants were reviewed and approved by Beijing Youan Hospital Research Ethics Committee. The patients/participants provided their written informed consent to participate in this study.

## Author Contributions

XL, XZ, HX, and BS conceived the study, ZL, HX, RW, YJ, ZYL, LY, and XW performed experiments, XL and XZ analyzed data, WX and HW selected patients, XL, XZ, AKLC, and BS wrote the manuscript, XL, CM, TZ, and BS revised the manuscript, and BS supervised the whole study. All authors contributed to the article and approved the submitted version.

## Funding

This work was supported by the Beijing Municipal Administration of Hospitals' Program (QML20181702 to XL, DFL20191701 to TZ), National Natural Science Foundation of China (NSFC, 81974303 and 81772165 to BS, 82072294 to ZL, 82072271 to TZ, 82002136 to HX), Health and Medical Research Fund (#18170032 to AKLC), the NSFC-NIH Biomedical collaborative research program (81761128001 to HW), the Beijing Municipal of Science and Technology Major Project (D161100000416003 to HW), and Beijing Key Laboratory for HIV/AIDS Research (BZ0089). The funders had no role in study design, data collection and analysis, decision to publish, or preparation of the manuscript.

## Conflict of Interest

The authors declare that the research was conducted in the absence of any commercial or financial relationships that could be construed as a potential conflict of interest.

## Publisher’s Note

All claims expressed in this article are solely those of the authors and do not necessarily represent those of their affiliated organizations, or those of the publisher, the editors and the reviewers. Any product that may be evaluated in this article, or claim that may be made by its manufacturer, is not guaranteed or endorsed by the publisher.

## Supplementary Material

Supplementary Figure 1 Correlation between the frequencies of B cell subsets and CD40L^+^cTfh cells in the AHI and CHI groups. **(A)**. The frequency of CD40L^+^cTfh cells is elevated in AHI (n=25) and CHI (n=25). All data shown are means ± SD. **(B–H)**. Correlation analysis of the CD40L^+^cTfh cell frequency with bulk B cells, AM, CM, NM, RM, TLM, and PB in the AHI and CHI groups.

Supplementary Figure 2 cTfh cells are activated after HIV-1 acute infection. **(A)**. Comparison of Ki-67 expression on bulk cTfh cells among HC (n=21), AHI (n=25), and CHI (n=25) groups. All data shown are means ± SD. Association analysis between the frequency of Ki-67^+^cTfh cells and that of ICOS^+^cTfh cells **(B)** or CD40L^+^cTfh cells **(C)** in the AHI and CHI groups.

Supplementary Figure 3 IL-21^+^cTfh cells are not responsible for abnormal B cell differentiation during acute or chronic HIV-1 infection stage. Comparison of plasma IL-21 concentration **(A)** and IL-21^+^cTfh cell proportion **(B)** among HC (n=21), AHI (n=25), and CHI (n=25) groups. All data shown are means ± SD. **(C)**. Association analysis between the frequency of IL-21^+^cTfh cells and plasma IL-21 concentration in the AHI and CHI groups. **(D–J)**. Correlation analysis of the IL-21^+^cTfh cell frequency with bulk B cells, AM, CM, NM, RM, TLM, and PB in the AHI and CHI groups.
